# Development and validation of a RP-HPLC method for simultaneous determination of five COVID-19 antiviral drugs in pharmaceutical formulations

**DOI:** 10.1038/s41598-025-09904-0

**Published:** 2025-07-15

**Authors:** Mohamed W. Nassar, Ahmed Serag, Mohamed A. Hasan, Mohamed Kamel

**Affiliations:** Pharmaceutical Analytical Chemistry Department, Faculty of Pharmacy, Al- Azhar University, Nasr City, 11751 Cairo Egypt

**Keywords:** Analytical chemistry, Green chemistry

## Abstract

A rapid, sensitive, and selective reversed-phase high-performance liquid chromatography (RP-HPLC) method was developed and validated for the simultaneous determination of five COVID-19 antiviral drugs: favipiravir, molnupiravir, nirmatrelvir, remdesivir, and ritonavir. The chromatographic separation was achieved on a Hypersil BDS C18 column (4.5 × 150 mm, 5 μm particle size) using an isocratic mobile phase consisting of water and methanol (30:70 v/v, pH 3.0 adjusted with 0.1% ortho-phosphoric acid) at a flow rate of 1 mL/min with UV detection at 230 nm. The optimized method demonstrated good chromatographic resolution with retention times of 1.23, 1.79, 2.47, 2.86, and 4.34 min for favipiravir, molnupiravir, nirmatrelvir, remdesivir, and ritonavir, respectively. The method was validated according to ICH guidelines, showing linearity in the concentration range of 10–50 µg/mL with correlation coefficients (r²) ≥ 0.9997 for all analytes. The limits of detection were 0.415–0.946 µg/mL, while the limits of quantification were 1.260–2.868 µg/mL. The method demonstrated high trueness (99.59-100.08%) and precision (RSD < 1.1%). The validated method was successfully applied to the determination of these drugs in their pharmaceutical formulations, with recovery values ranging from 99.98 to 100.7% and no significant interference from excipients. Comprehensive greenness and practicality evaluations using five assessment tools yielded favorable scores: AGREE (0.70), AGREEprep (0.59), MoGAPI (70%), BAGI (82.5), and CACI (79), indicating good environmental performance and excellent practical applicability for routine pharmaceutical quality control analysis. The multi-tool assessment confirmed the method’s environmental friendliness through strategic solvent selection and minimal sample preparation requirements, while demonstrating superior practical implementation characteristics including cost-effectiveness and accessibility in standard analytical laboratories.

## Introduction

The COVID-19 pandemic has driven rapid development and repurposing of antiviral medications, resulting in several therapeutic options receiving emergency use authorization from regulatory authorities^1,2^. Among these, favipiravir (FVP), molnupiravir (MLP), nirmatrelvir (NTV), remdesivir (RMD), and ritonavir (RTN) have emerged as important treatment options, either as monotherapy or in combination regimens^3,4^.

These antivirals represent diverse structural classes with distinct mechanisms of action. Favipiravir and molnupiravir are nucleoside analogs that interfere with viral RNA replication^5,6^. Remdesivir, an adenosine analog prodrug, inhibits viral RNA-dependent RNA polymerase^7^. The combination of nirmatrelvir (a viral protease inhibitor) with ritonavir (a pharmacokinetic enhancer) represents another therapeutic approach for managing COVID-19^8,9^.

The structural complexity and diverse physicochemical properties of these compounds present significant analytical challenges. Several individual analytical methods have been reported for these drugs, including HPLC^10–16^, LC-MS/MS^17–20^, spectroscopy^21–29^, and electrochemical techniques^30–32^. However, most published methods focus on the analysis of individual drugs or binary combinations. While these methods provide valuable analytical approaches, they necessitate multiple separate analyses when monitoring multiple drugs, increasing analysis time, resource utilization, and cost.

While spectrophotometric methods offer advantages including simplicity, cost-effectiveness, and widespread availability, they face significant limitations for simultaneous multi-component analysis due to spectral overlap and matrix interferences^33–37^. HPLC methods, despite higher instrumental costs and complexity, provide superior selectivity through chromatographic separation, enabling accurate quantification of structurally similar compounds without derivatization^38^. For complex pharmaceutical formulations containing multiple active ingredients, HPLC offers unmatched specificity and the ability to resolve matrix interferences that would compromise spectrophotometric determinations^39^. However, HPLC methods typically require longer analysis times, higher solvent consumption, and specialized expertise, making the choice between techniques dependent on analytical requirements, sample complexity, and available resources. Simultaneous determination methods offer significant advantages for quality control laboratories, pharmacokinetic studies, and clinical monitoring. Despite the importance of these antivirals in COVID-19 management, our literature review revealed no published method for their simultaneous quantification. Such a method would be particularly valuable for: (1) quality control laboratories analyzing multiple COVID-19 therapeutics, (2) clinical studies investigating combination therapies, and (3) wastewater monitoring studies tracking multiple antivirals.

Therefore, this study aimed to develop and validate a simple, sensitive, and selective RP-HPLC method for the simultaneous determination of favipiravir, molnupiravir, nirmatrelvir, remdesivir, and ritonavir in both pure form and pharmaceutical formulations. The specific objectives include: (1) Development of an optimized chromatographic method enabling baseline separation of favipiravir, molnupiravir, nirmatrelvir, remdesivir, and ritonavir within a reasonable analysis time; (2) Comprehensive validation according to ICH guidelines including linearity, trueness, precision, specificity, robustness, and detection limits; (3) Assessment of method greenness using multiple evaluation tools (AGREE, AGREEprep, MoGAPI) and practical applicability using (BAGI, CACI); and (4) Application to pharmaceutical formulations to demonstrate real-world utility. This represents the first validated analytical procedure for the simultaneous determination of these five COVID-19 antivirals, offering significant advantages in terms of time, resource utilization, and analytical efficiency.

## Experimental

### Materials and chemicals

Pure reference standards of nirmatrelvir (99.36%) and ritonavir (99.62%) were kindly supplied by Pfizer, Inc., Egypt. Paxlovid tablets, containing pink oval nirmatrelvir film-coated tablets (150 mg) co-packaged with white ritonavir film-coated tablets (100 mg), were obtained from Pfizer, Inc., Egypt. The recommended dose is two tablets of nirmatrelvir plus one tablet of ritonavir.

Pure reference standards of favipiravir (99.55%), molnupiravir (98.86%) and remdesivir (99.29%) were kindly supplied by EVA-PHARMA, Giza, Egypt. Avipiravir tablets containing favipiravir (200 mg), Molnupiravir-Eva Pharma capsules containing molnupiravir (200 mg), and Remdesivir-Eva Pharma powder for intravenous infusion containing remdesivir (100 mg) were supplied by Eva Pharma, Giza, Egypt.

Methanol (HPLC grade, ≥ 99.9% purity) was purchased from Sigma Aldrich (Germany). Ortho-phosphoric acid (analytical grade, 85%) was obtained from El-Nasr Pharmaceutical Chemicals Company (Egypt). Water used throughout the procedure was freshly distilled and filtered through a 0.45 μm membrane filter. Whatman filter paper No. 41 (pore size 20–25 μm, Whatman International Ltd., England) was used for filtration. All reference standards were stored at 2–8 °C in tightly closed containers protected from light. Standard solutions were freshly prepared on the day of analysis.

### Instruments

HPLC analysis was performed using an Agilent 1260 Infinity II system (Agilent Technologies, USA) equipped with a quaternary solvent delivery pump (G7111B), an autosampler (G7129A) with 100 µL injection capacity, a thermostatted column compartment (G7116A) maintained at 25 ± 0.5 °C, and a diode array detector (G7115A) with a 10 mm path length flow cell and wavelength range of 190–800 nm. Chromatographic data acquisition and processing were performed using Agilent ChemStation software (Agilent Technologies, USA). A digital pH meter (Jenway, model 3510, UK) was used for pH measurements. An ultrasonic bath (Elma S100H, Germany) was used for degassing the mobile phase and dissolving the samples.

### Standard solutions

#### Preparation of stock standard solutions

Stock standard solutions of favipiravir, molnupiravir, nirmatrelvir, remdesivir, and ritonavir (1000 µg/mL each) were prepared separately by accurately weighing 100 mg of each reference standard into separate 100 mL volumetric flasks. Approximately 70 mL of methanol was added to each flask, and the contents were sonicated for 10 min to ensure complete dissolution. The solutions were then allowed to cool to room temperature, and the volumes were made up to the mark with methanol. These stock solutions were stored at 4 °C and were stable for at least two weeks.

#### Preparation of working standard solutions

Working standard solutions (100 µg/mL) were prepared daily by diluting 10 mL of each stock solution to 100 mL with methanol. Further dilutions were made with methanol to obtain the required concentrations for analysis.

### Chromatographic conditions

Isocratic chromatographic separation was performed on a Hypersil BDS C18 column (150 mm × 4.6 mm; 5 μm particle size) maintained at 25 ± 2 °C. The mobile phase consisted of water and methanol in the ratio of 30:70% v/v, with pH adjusted to 3.0 using 0.1% ortho-phosphoric acid. The mobile phase was filtered through a 0.45 μm membrane filter and degassed ultrasonically prior to use. The flow rate was maintained at 1.0 mL/min, and the injection volume was 20 µL. UV detection was performed at 230 nm. The total run time was 6 min. Before sample analysis, the column was equilibrated with the mobile phase for at least 30 min until a stable baseline was achieved.

### Procedures

#### Construction of calibration graphs

Accurately measured volumes (1, 2, 3, 4, and 5 mL) of each working standard solution (100 µg/mL) were transferred into five separate series of 10 mL volumetric flasks and diluted to volume with methanol to obtain final concentrations of 10, 20, 30, 40, and 50 µg/mL for each drug.

Each calibration level was prepared in triplicate. A 20 µL aliquot of each solution was injected into the HPLC system under the optimized chromatographic conditions. The chromatograms were recorded, and the peak areas were measured. Calibration curves were constructed by plotting the mean peak areas versus the corresponding drug concentrations in µg/mL. The regression equations were calculated for each analyte.

#### Analysis of laboratory-prepared mixtures

To evaluate the selectivity and trueness of the method for simultaneous determination, laboratory-prepared mixtures containing the five drugs in different concentration ratios were analyzed. Aliquots of standard solutions (100 µg/mL) of favipiravir, molnupiravir, nirmatrelvir, remdesivir, and ritonavir were transferred into a series of 10 mL volumetric flasks, keeping the ratio between them as in the common dosage forms (2:2:1.5:1:1 v/v, respectively).

The solutions were diluted to volume with methanol and mixed thoroughly. A 20 µL aliquot of each mixture was injected in triplicate and analyzed under the optimized chromatographic conditions. The concentration of each drug was calculated using the corresponding regression equation.

#### System suitability tests

System suitability tests were performed to verify that the chromatographic system was adequate for the analysis. The parameters evaluated included retention time, retention factor, theoretical plates, tailing factor, and resolution factor. The acceptance criteria were: theoretical plates > 2000, tailing factor ≤ 2, and resolution factor > 2 between adjacent peaks.

#### Application to pharmaceutical preparations

Five Avipiravir tablets (200 mg favipiravir/tablet) were weighed and finely powdered. Similarly, the contents of five Molnupiravir-Eva Pharma capsules (200 mg molnupiravir/capsule) were emptied and mixed thoroughly. Five Paxlovid tablets of nirmatrelvir (150 mg/tablet) and five tablets of ritonavir (100 mg/tablet) were separately weighed and finely powdered.

From each powdered sample, an amount equivalent to 10 mg of the respective drug was accurately weighed and transferred to a 100 mL volumetric flask. For remdesivir, the contents of five vials of Remdesivir-Eva Pharma (100 mg/vial) were reconstituted according to the manufacturer’s instructions, and a volume equivalent to 10 mg of remdesivir was transferred to the same 100 mL volumetric flask.

Approximately 70 mL of methanol was added to the flask, and the mixture was sonicated for 15 min to ensure complete extraction of the drugs. The solution was then filtered through Whatman filter paper No. 41, and the filtrate was collected in a 100 mL volumetric flask. The filter paper was washed with small portions of methanol, and the washings were added to the filtrate. The volume was completed to the mark with methanol to obtain a solution containing approximately 100 µg/mL of each drug.

This solution was further diluted with methanol to obtain a final solution containing approximately 30 µg/mL of each drug. A 20 µL aliquot was injected into the HPLC system and analyzed under the optimized chromatographic conditions. The concentration of each drug was calculated using the corresponding regression equation.

### Greenness and blueness assessment

The environmental impact of the developed analytical method was evaluated using established greenness and blueness assessment tools.

#### AGREE assessment

The Analytical GREEnness (AGREE) metrics were calculated according to the procedure described by Pena-Pereira et al.^40^. The method was evaluated based on 12 principles of green analytical chemistry, including direct analytical eco-scale score, sample collection and preparation, reagents/solvents used, energy consumption, waste generation, and operator safety. Each principle was assigned a score between 0 and 1, and the final AGREE value was calculated.

#### AGREEprep assessment

The AGREEprep metric was employed to specifically evaluate the greenness of sample preparation procedures according to the ten principles of green sample preparation. The assessment was conducted using the AGREEprep software available at mostwiedzy.pl/AGREEprep, evaluating criteria including sample preparation placement, use of safer solvents, sustainable materials, waste minimization, sample economy, throughput maximization, step integration, energy consumption, post-preparation configuration, and operator safety^41^.

#### MoGAPI assessment

The Modified Green Analytical Procedure Index (MoGAPI) was applied to provide both visual assessment and quantitative scoring of method greenness. The evaluation utilized the MoGAPI software (bit.ly/MoGAPI) to assess sample preparation, reagent usage, instrumentation, and waste parameters, generating both a numerical score and color-coded pictogram representation^42^.

#### BAGI assessment

The blue applicability grade index (BAGI) was determined according to the methodology described by Manousi et al.^43^. This comprehensive assessment evaluated the practicality and applicability of the developed analytical method based on ten key attributes: (1) type of analysis, (2) number of analytes simultaneously determined, (3) analytical technique and instrumentation, (4) number of samples that can be simultaneously treated, (5) sample preparation methodology, (6) number of samples analyzed per hour, (7) type of reagents and materials used, (8) requirement for preconcentration, (9) degree of automation, and (10) sample amount required.

Each attribute was scored on a scale from 2.5 to 10 points, with higher scores indicating better practical applicability. The assessment generated both a visual asteroid pictogram representation and a numerical score ranging from 25 to 100.

#### CACI assessment

The Click Analytical Chemistry Index (CACI) was employed to evaluate method practicality and cost-effectiveness using the CACI software (bit.ly/CACI2025). The assessment covered eight criteria including sample size, sample preparation complexity, feasibility, application scope, portability, automation level, sensitivity, and analysis time^44^.

## Results and discussion

### Method development and optimization

The primary objective of this work was to develop a simple, sensitive, and selective RP-HPLC method for the simultaneous determination of five COVID-19 antiviral drugs. Method development followed a systematic approach to achieve optimal separation with reasonable analysis time.

#### Selection of detection wavelength

**Fig. 1:**
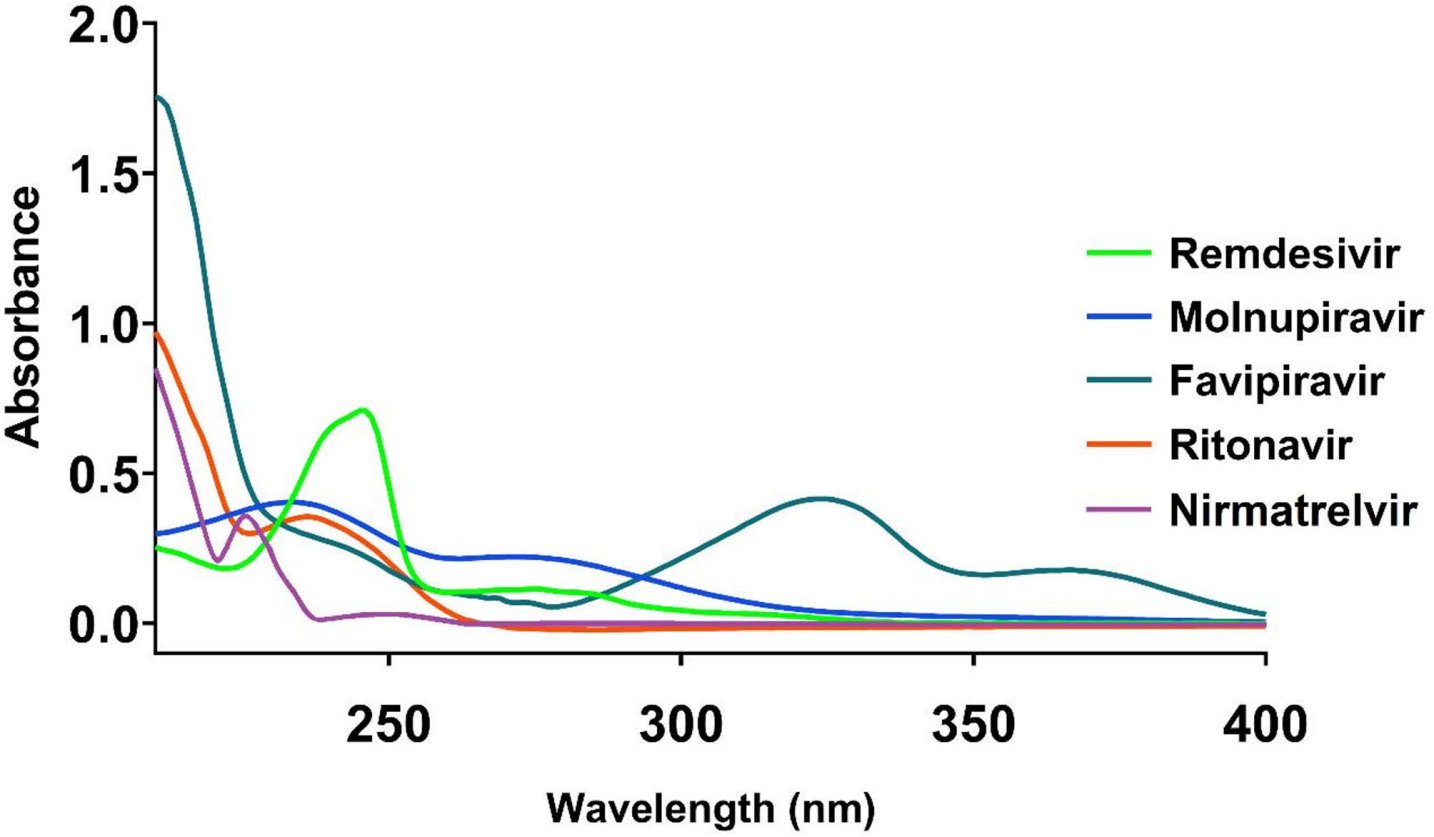
UV absorption spectra of favipiravir, molnupiravir, remdesivir, ritonavir and nirmatrelvir in the wavelength range of 210–400 nm.

UV absorption spectra were recorded for each drug in the range of 200–400 nm (Fig. 1). The spectra revealed distinctive absorption profiles for each compound. Favipiravir exhibited a strong absorption below 220 nm and a characteristic peak at approximately 320 nm. Molnupiravir showed relatively consistent absorption across the 210–270 nm range with diminishing absorption at higher wavelengths. Remdesivir demonstrated a prominent absorption maximum at approximately 245 nm, while ritonavir showed strong absorption below 240 nm that rapidly decreased at higher wavelengths. Notably, nirmatrelvir exhibited maximum absorption at approximately 215 nm.

After evaluating several wavelengths, 230 nm was selected as the optimum detection wavelength as it provided adequate sensitivity for all five compounds with minimal interference from mobile phase components. At this wavelength, all compounds showed sufficient absorbance to enable sensitive detection while maintaining good signal-to-noise ratios across the concentration range of interest.

#### Optimization of chromatographic conditions

Several chromatographic parameters were carefully evaluated to achieve optimal separation of the five drugs with good resolution and reasonable analysis time.

##### Column selection

Initial trials were conducted using different C18 columns (Symmetry C18, Eclipse XDB-C18, and Hypersil BDS C18) with different dimensions. The Hypersil BDS C18 column (150 mm × 4.6 mm, 5 μm) demonstrated superior performance in terms of peak shape, resolution, and column efficiency for all five compounds.

##### Mobile phase composition

The selection of the mobile phase composition was critical for achieving adequate separation with reasonable analysis time. Various combinations of organic modifiers (methanol and acetonitrile) with water were evaluated. While acetonitrile provided slightly better peak shapes, methanol was selected due to its lower toxicity, cost-effectiveness, and sufficient separation capability, aligning with green analytical chemistry principles.

Different proportions of methanol (60:40, 65:35, 70:30, 75:25, and 80:20 v/v) were investigated. At lower methanol concentrations (60–65%), the analysis time was excessively long with poor resolution between nirmatrelvir and remdesivir. With higher methanol concentrations (75–80%), favipiravir eluted too close to the void volume, and the resolution between nirmatrelvir and remdesivir decreased. The ratio of 70:30 v/v (methanol) provided the optimal balance, achieving good resolution between all adjacent peaks with a reasonable analysis time of less than 5 min.

##### Effect of pH

The effect of mobile phase pH on separation was studied in the range of 2.5–4.5, adjusted using ortho-phosphoric acid. At pH values above 4.0, peak tailing was observed for remdesivir and ritonavir. At pH values below 2.5, while silanol groups remain largely non-ionized potentially improving peak shapes for basic compounds, the column stability was compromised due to increased risk of silica dissolution, leading to decreased column efficiency over time. A pH of 3.0 was found to be optimal, providing symmetrical peak shapes and excellent resolution between all compounds.

##### Flow rate optimization

Flow rates between 0.8 and 1.2 mL/min were evaluated to optimize the analysis time while maintaining resolution. At lower flow rates (0.8 mL/min), the analysis time was extended without significant improvement in resolution. At higher flow rates (1.2 mL/min), a slight decrease in resolution between nirmatrelvir and remdesivir was observed. A flow rate of 1.0 mL/min was selected as the optimal compromise, providing adequate resolution with reasonable analysis time.

**Fig. 2:**
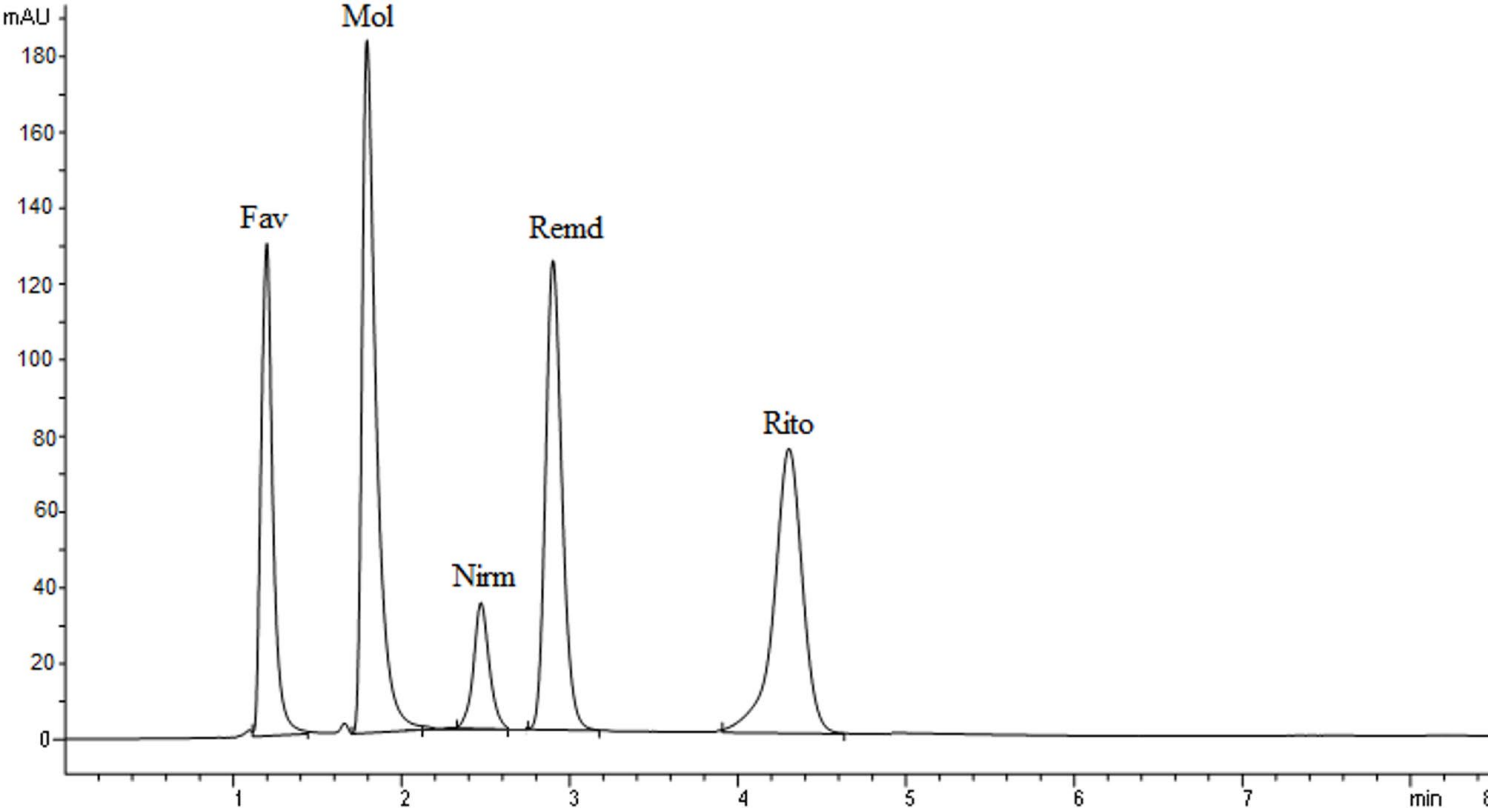
HPLC Chromatogram of Favipiravir (10 µg/ml), Molnupiravir (10 µg/ml) Nirmatrelvir (10 µg/ml), Remdesivir (10 µg/ml) and Ritonavir (10 µg/ml) using water and methanol in the ratio of (30:70% v/v) and pH 3 maintained by 0.1% ortho phosphoric acid as a mobile phase with UV detection at 230 nm.

Under the optimized conditions, the five drugs were well-separated with retention times of 1.23, 1.79, 2.47, 2.86, and 4.34 min for favipiravir, molnupiravir, nirmatrelvir, remdesivir, and ritonavir, respectively, as shown in Fig. 2. The total analysis time was less than 5 min, making the method suitable for routine analysis.

### Method validation

The developed method was validated according to ICH guidelines for specificity, linearity, range, trueness, precision, limits of detection and quantification, and robustness^45^.

#### Linearity and range

**Fig. 3:**
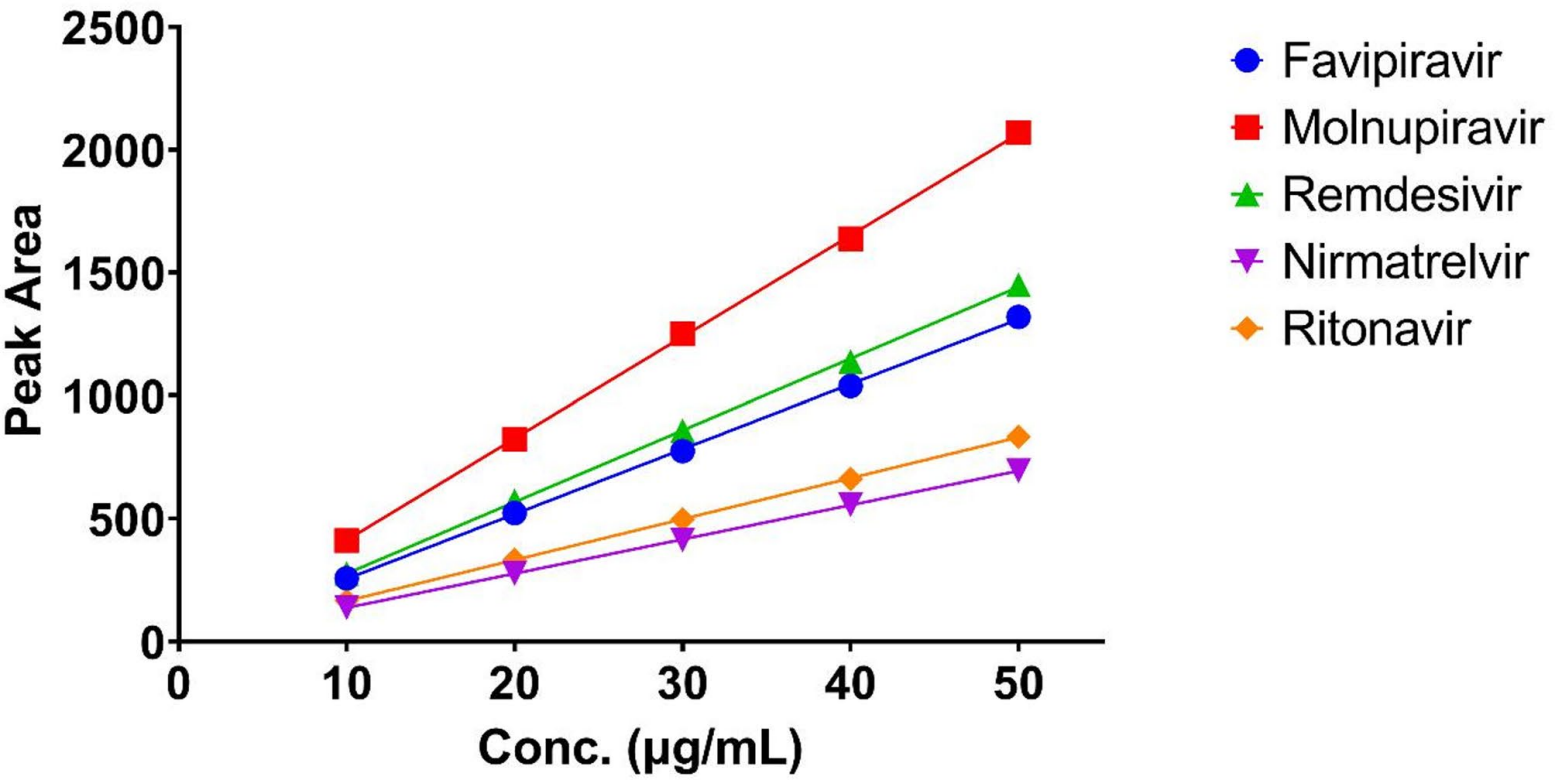
Calibration curves showing the relationship between peak area and concentration (10–50 µg/mL) for the five COVID-19 antiviral drugs. All compounds demonstrated excellent linearity with correlation coefficients (r²) greater than 0.999. The different slopes reflect the varying detector responses at 230 nm, with molnupiravir showing the highest sensitivity and nirmatrelvir the lowest.

Linearity was evaluated by analyzing five concentration levels (10–50 µg/mL) for each drug in triplicate. The calibration curves were constructed by plotting the peak areas against the corresponding concentrations (Fig. 3). The regression equations, correlation coefficients, ranges, slopes, and intercepts are presented in Table 1.

**Table 1:** Regression and validation parameters of the proposed HPLC method.

Parameters		Favipiravir	Molnupiravir	Nirmatrelvir	Remdesivir	Ritonavir
Wavelength (nm)		230	230	230	230	230
Linearity range (µg/ml)		10 ─ 50	10 ─ 50	10 ─ 50	10 ─ 50	10 ─ 50
- Slope (b) - Intercept (a)		26.3796 − 8.6493	41.247 0.0259	13.929 − 1.576	29.116 − 14.512	16.647 − 0.748
Coefficient of determination(r^2^)		0.9997	0.9998	0.9999	0.9997	0.9999
LOD (µg/ml)		0.946	0.747	0.415	0.877	0.470
LOQ (µg/ml)		2.868	2.264	1.260	2.657	1.424
Trueness (%R)*		99.59 ± 0.82	99.87 ± 0.671	100.08 ± 0.54	100.05 ± 0.71	100.08 ± 0.47
Precision (%RSD)**	Repeatability	0.901	0.720	0.606	0.949	0.832
	Intermediate precision	1.076	0.806	0.770	0.995	0.731

*Average of nine determinations (triplicate determination of three concentration level).

**%RSD of %Recovery of nine determinations (triplicate determination of three concentration level).

All five drugs showed excellent linearity in the concentration range of 10–50 µg/mL with correlation coefficients (r²) greater than 0.999, indicating excellent linearity. The high values of correlation coefficients and the low values of intercepts demonstrate the linearity of the method over the studied concentration range.

#### Limits of detection and quantification

The limits of detection (LOD) and quantification (LOQ) were calculated based on the standard deviation of the response and the slope of the calibration curve using the formulas: LOD = 3.3σ/S and LOQ = 10σ/S, where σ is the standard deviation of the y-intercept and S is the slope of the calibration curve.

As shown in Table 1, the LOD values ranged from 0.415 to 0.946 µg/mL, and the LOQ values ranged from 1.260 to 2.868 µg/mL, indicating the high sensitivity of the developed method for all five drugs.

#### Trueness

The trueness of the method was assessed by analyzing laboratory-prepared mixtures containing known amounts of the five drugs at three concentration levels (10, 30, and 50 µg/mL) in triplicate. The percentage recoveries ranged from 99.59 to 100.08% with low standard deviations (Table 1), confirming the high trueness of the method.

#### Precision

##### Repeatability (Intra-day Precision)

Repeatability was evaluated by analyzing three different concentrations (10, 30, and 50 µg/mL) of each drug in triplicate on the same day. The RSD values were less than 1.0% for all drugs (Table 1), indicating excellent repeatability.

##### Intermediate precision (Inter-day precision)

Intermediate precision was assessed by analyzing three different concentrations (10, 30, and 50 µg/mL) of each drug in triplicate on three consecutive days. The RSD values were less than 1.1% for all drugs (Table 1), demonstrating the high intermediate precision of the method.

#### Specificity

The specificity of the method was evaluated by analyzing laboratory-prepared mixtures containing the five drugs in different ratios and comparing them with the individual standard solutions. No interference was observed between the five drugs, and all peaks were well-resolved with resolution factors greater than 2.0 between adjacent peaks.

Additionally, the specificity was confirmed by applying the standard addition technique to pharmaceutical formulations. Known amounts of pure standards were added to pre-analyzed pharmaceutical formulations, and the mixtures were analyzed. The percentage recoveries ranged from 97.65 to 102.46% (Table 2), indicating that excipients in the pharmaceutical formulations did not interfere with the determination of the active ingredients.

**Table 2:** Quantitative determination of favipiravir, molnupiravir, nirmatrelvir, Remdesivir and Ritonavir in its pharmaceutical dosage form by HPLC method using standard addition technique:

Drug	Pharmaceutical taken(µg/ml)	Pharmaceutical found^*^ (µg/ml)	Pure added (µg/ml)	Pure found ^**^ ( µg/ml)	%Recovery
Favipiravir	10	10.10	10	10.10	101.04
			20	19.97	99.87
			30	30.19	100.65
Mean±%RSD					100.52 ± 0.592
Molnupiravir	10	9.94	10	10.03	100.39
			20	19.98	99.90
			30	29.29	97.65
Mean±%RSD					99.31 ± 1.47
Nirmatrelvir	10	9.98	10	10.13	101.35
			20	20.04	100.24
			30	29.59	98.63
Mean±%RSD					100.07 ± 1.36
Remdesivir	10	9.918	10	10.24	102.46
			20	19.83	99.19
			30	30.07	100.25
Mean±%RSD					100.63 ± 1.66
Ritonavir	10	10.21	10	9.86	98.62
			20	20.14	100.73
			30	30.12	100.42
Mean±%RSD					99.92 ± 1.14

* Average of five experiments.

** Average of three experiments.

#### System suitability

System suitability parameters, including retention time, retention factor, theoretical plates, tailing factor, and resolution factor, were evaluated to ensure the adequacy of the chromatographic system. Most results, presented in Table 3, met the acceptance criteria^46^, confirming the suitability of the system for the intended analysis. Despite favipiravir’s early elution (K’ = 0.12), the compound demonstrated excellent chromatographic behavior with symmetric peak shape and no interference from void volume components. The retention factor progression (0.12 → 2.95) reflects the increasing lipophilicity from the highly polar favipiravir to the most hydrophobic ritonavir, confirming appropriate reversed-phase selectivity. The total analysis time was less than 5 min, making the method suitable for high-throughput routine analysis.

**Table 3:** System suitability results for the determination of favipiravir, molnupiravir, nirmatrelvir, Remdesivir and Ritonavir by the proposed HPLC method.

Parameters	Favipiravir	Molnupiravir	Nirmatrelvir	Remdesivir	Ritonavir	Reference value ^46^
Retention time (t_*R*_)	1.23 min	1.79 min	2.47 min	2.86 min	4.34 min	–
Retention factor (K´)	0.12	0.63	1.25	1.60	2.95	1–10
Theoretical Plates (N)	8236	9468	3629	6722	4047	2000≤
Tailing factor (T)	1.3	1.5	0.9	1.1	1.7	2≥
Resolution factor (Rs)	2.7		2.4	2.1	4.3	2≤

#### Robustness

The robustness of the method was assessed by making deliberate small changes in the chromatographic conditions, including flow rate (± 0.1 mL/min) and mobile phase composition (± 2% methanol). The results, summarized in Table 4, show that these small changes did not significantly affect the retention times or tailing factors, confirming the robustness of the method.

**Table 4:** Robustness results for the determination of favipiravir, molnupiravir, nirmatrelvir, Remdesivir and Ritonavir by the proposed HPLC procedure.

Parameters		Retention time (t_R_)					Tailing factor (T)				
		Fav	Moln	Nirm	Remd	Rito	Fav	Moln	Nirm	Remd	Rito
Flow rate (ml/min.)	0.9	1.25	1.84	2.49	2.92	4.41	1.35	1.53	0.99	1.16	1.77
	1	1.23	1.79	2.47	2.86	4.34	1.33	1.51	0.97	1.15	1.74
	1.1	1.22	1.75	2.41	2.83	4.32	1.32	1.51	0.96	1.12	1.73
Mobile phase ratio (water: methanol)	32:68	1.24	1.79	2.45	2.84	4.35	1.34	1.52	0.96	1.14	1.75
	30:70	1.23	1.79	2.47	2.86	4.34	1.33	1.51	0.97	1.15	1.74
	28:72	1.21	1.78	2.48	2.87	4.33	1.32	1.51	0.99	1.15	1.73

### Greenness and blueness assessment

#### AGREE assessment

**Fig. 4:**
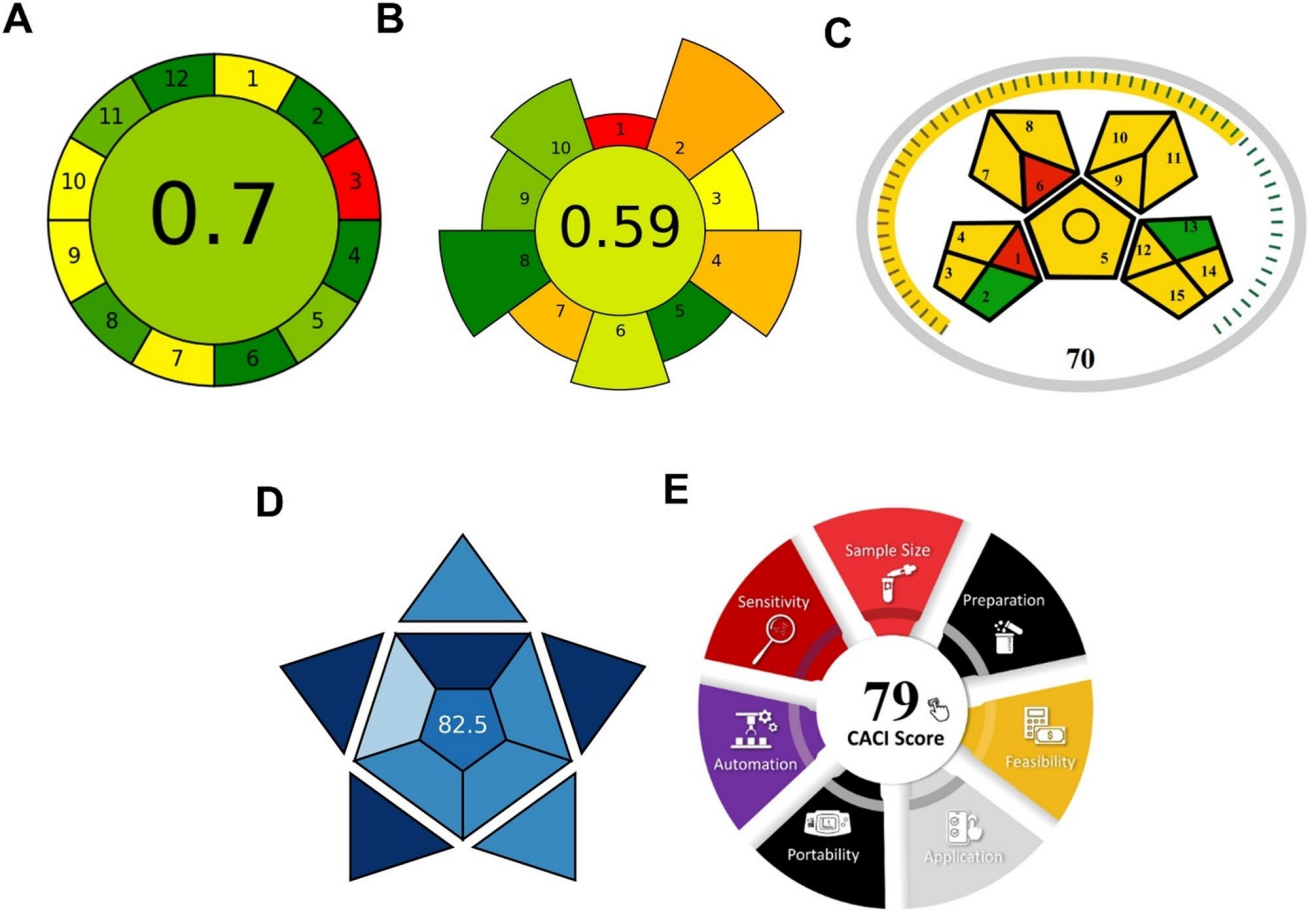
Comprehensive green and blue assessment of the developed RP-HPLC method using five evaluation tools: (**A**) AGREE assessment (score: 0.7) showing overall method greenness across 12 principles of green analytical chemistry; (**B**) AGREEprep assessment (score: 0.59) for sample preparation greenness; (**C**) MoGAPI assessment (score: 70%) with pentagram visualization of method greenness categories; (**D**) BAGI assessment (score: 82.5) evaluating practical applicability across ten implementation attributes; (**E**) CACI assessment (score: 79) measuring cost-effectiveness and accessibility.

The greenness of the developed method was evaluated using the AGREE metrics. The method achieved an AGREE score of 0.70 out of 1, indicating a high level of greenness (Fig. 4A). The use of methanol instead of more toxic solvents, the isocratic elution mode, the moderate flow rate, and the absence of sample derivatization contributed positively to the AGREE score.

The main factors affecting the greenness score were the use of organic solvent (methanol) in the mobile phase and the energy consumption of the HPLC system. However, these are inherent limitations of HPLC techniques and were minimized by optimizing the method to achieve rapid analysis with reasonable solvent consumption.

#### AGREEprep assessment

The AGREEprep evaluation, specifically designed for sample preparation assessment, achieved a score of **0.59**, reflecting the ten principles of green sample preparation (Fig. 4B). This assessment provides targeted evaluation of the sample treatment steps that are often the most resource-intensive component of analytical methods. The assessment highlighted several environmentally favorable aspects of our sample preparation approach. The method employs ex situ sample preparation with minimal transportation requirements, reducing the overall environmental footprint. The absence of complex derivatization reactions eliminates the need for additional chemical reagents and reduces waste generation. Sample preservation requirements are minimal, requiring only standard laboratory storage conditions without specialized preservation chemicals. The AGREEprep analysis identified specific areas where the sample preparation protocol could be further optimized for environmental sustainability. The current extraction scale operates at conventional levels rather than implementing microscale approaches that could reduce solvent consumption. Energy consumption associated with ultrasonic dissolution and filtration steps represents an opportunity for optimization through alternative sample treatment techniques. The integration of sample preparation steps could be enhanced to reduce the number of separate operations and associated waste generation. Despite these optimization opportunities, the AGREEprep score of 0.59 confirms that our sample preparation approach follows sustainable practices while maintaining the simplicity and robustness essential for routine pharmaceutical quality control. The evaluation demonstrates that even straightforward extraction procedures can achieve acceptable green performance when properly designed, supporting the principle that effective green analytical chemistry does not necessarily require complex or exotic sample preparation techniques.

#### MoGAPI assessment

The MoGAPI analysis provided a comprehensive score of **70%**, confirming acceptable greenness performance through systematic evaluation of 15 distinct criteria organized across five pentagram sectors (Fig. 4C). This visual assessment tool combines quantitative scoring with intuitive pictogram representation, providing immediate identification of method strengths and environmental impact areas. The sample preparation sector demonstrated favorable performance (green/yellow-green) due to straightforward offline collection, minimal preservation requirements, and simple dissolution procedures without complex extraction techniques. The instrumentation sector reflected moderate performance (yellow), primarily influenced by inherent HPLC energy consumption, though mitigated by short analysis time and isocratic elution reducing equilibration requirements. Waste generation achieved acceptable performance with well-controlled methanol-water waste and no hazardous waste requiring specialized disposal. The score of 70% places the method in the ‘acceptable green’ category when compared to typical pharmaceutical HPLC methods. The balanced pentagram shape indicates uniform environmental performance without critical weaknesses, while yellow coloring in energy sectors reflects inherent technique limitations rather than method-specific deficiencies, providing clear guidance for future optimization strategies.

#### BAGI assessment

The Blue Applicability Grade Index (BAGI) was used to evaluate the practicality and applicability of the developed method. The method achieved a BAGI score of 82.5 out of 100, indicating excellent practical applicability for routine analysis (Fig. 4D). This high score reflects the method’s strengths across multiple dimensions of laboratory practicality. The method provides quantitative and confirmatory analysis of five compounds from different chemical classes using simple instrumentation that is readily available in most analytical laboratories. Sample preparation is straightforward, requiring only basic extraction techniques without complex derivatization or preconcentration steps. The analysis throughput is efficient, allowing for 5–10 samples to be processed per hour including sample preparation time. Furthermore, the method utilizes only common, commercially available reagents, enhancing its accessibility and cost-effectiveness. The semi-automated nature of the analytical procedure with standard HPLC equipment reduces operator intervention while maintaining flexibility. Sample requirements are minimal, which conserves valuable pharmaceutical materials. These practical advantages, quantified by the high BAGI score, demonstrate that the developed method is not only environmentally friendly but also highly practical for implementation in pharmaceutical quality control laboratories with typical resources and instrumentation.

#### CACI assessment

The CACI assessment achieved a score of **79**, reflecting good practical applicability with particular emphasis on cost-effectiveness and implementation accessibility (Fig. 4E). The evaluation covered eight criteria specifically designed for analytical method practicality: sample size requirements (moderate performance due to mL-level volumes), sample preparation complexity (good score for minimal preparation steps), feasibility (excellent score for commercial reagent availability and standard instrumentation), application scope (good performance for quantitative pharmaceutical analysis), portability (moderate score for benchtop instrumentation), automation level (moderate score for semi-automated operation), sensitivity (good performance for pharmaceutical concentration ranges), and analysis time (excellent score for sub-5-minute runtime). The assessment confirmed that the method requires reasonable resources for implementation, uses cost-effective reagents (estimated <$10 per sample), and provides excellent time efficiency. The CACI evaluation particularly highlighted the method’s strength in practical applicability for laboratories with standard analytical capabilities.”

#### Integrated green and blue profile analysis

The comprehensive five-tool assessment approach provides a holistic evaluation framework that addresses both environmental sustainability and practical implementation considerations. The green assessment tools (AGREE: 0.7, AGREEprep: 0.59, MoGAPI: 70%) collectively confirm good environmental performance with scores ranging in the acceptable-to-good range. The convergent results across green metrics identify consistent strengths in solvent selection and reagent safety, while highlighting energy consumption as a common area for improvement across all evaluations.

The blue assessment tools (BAGI: 82.5, CACI: 79) demonstrate excellent practical applicability, with both metrics confirming the method’s suitability for routine laboratory implementation. The high blue scores reflect the method’s strengths in accessibility, cost-effectiveness, and technical feasibility, addressing critical factors for widespread adoption in pharmaceutical quality control laboratories.

When compared to individual analytical methods reported in the literature, our simultaneous approach demonstrates superior resource efficiency. Individual HPLC methods for these compounds typically require 10–15 min per analysis, consuming 15–25 mL of organic solvents per sample. Our simultaneous method reduces total analysis time by approximately 75% and decreases overall solvent consumption by 60% when analyzing all five compounds, representing significant improvements in both environmental impact and economic efficiency. The multi-tool evaluation reveals that while the method achieves good overall sustainability performance, the primary limitation lies in the inherent energy consumption of HPLC instrumentation. Future optimization strategies should focus on method miniaturization, alternative detection systems, or implementation of energy-efficient chromatographic conditions to further enhance the environmental profile. The integrated assessment positions our method as a practical and environmentally conscious analytical solution that balances regulatory compliance requirements with sustainability considerations. The convergent positive results across all five assessment tools provide robust evidence for the method’s suitability for routine pharmaceutical analysis while maintaining awareness of environmental impact.

### Application to pharmaceutical formulations

The validated method was successfully applied to the determination of favipiravir, molnupiravir, nirmatrelvir, remdesivir, and ritonavir in their respective pharmaceutical formulations: Avipiravir tablets (200 mg favipiravir), Molnupiravir-Eva Pharma capsules (200 mg molnupiravir), Paxlovid tablets (150 mg nirmatrelvir and 100 mg ritonavir), and Remdesivir-Eva Pharma powder for intravenous infusion (100 mg remdesivir).

The assay results, presented in Table 5, showed high recovery percentages ranging from 99.98 to 100.7% with RSD values less than 1.23%, indicating the trueness and precision of the method for pharmaceutical formulation analysis. These results confirm that the common excipients present in the formulations did not interfere with the quantification of the active ingredients.

**Table 5:** Determination of Favipiravir, Molnupiravir, Nirmatrelvir, Remdesivir and Ritonavir in its pharmaceutical dosage form by the proposed HPLC method and the reported methods.

Parameters	Proposed HPLC method					Reported methods				
	Fav	Moln	Nirm	Remd	Rito	Fav	Moln	Nirm	Remd	Rito
Mean	100.32	100.2	100.7	100.5	99.98	100.1	99.81	99.77	99.73	100.07
%RSD	0.914	1.03	0.775	1.101	1.23	0.706	0.829	0.962	0.994	0.734
Number of measurements	5	5	5	5	5	5	5	5	5	5
Student’s t-test	0.381 (2.306)^*^	0.792 (2.306)^*^	1.717 (2.306)^*^	1.301 (2.306)^*^	1.321 (2.306)^*^	–	–	–	–	–
F-value	1.683 (6.388) ^*^	1.562 (6.388)^*^	1.095 (6.388)^*^	1.81 (6.388)^*^	1.523 (6.388)^*^	–	–	–	–	–

* The values in parenthesis are the tabulated values of “t “and “F” at (P = 0.05).

To further validate the method’s applicability, statistical comparison of the results was performed against those obtained by previously reported methods for the individual drugs^47–49^ using Student’s t-test and F-test at 95% confidence level. The calculated t and F values were less than the tabulated values (Table 5), indicating no significant difference between the proposed method and the reported methods in terms of trueness and precision.

These findings confirm that the developed method is suitable for routine quality control analysis of these five COVID-19 antivirals in their pharmaceutical formulations, offering the advantage of simultaneous determination with reduced analysis time and resource consumption compared to individual methods.

## Conclusion and future directions

This study presents the first validated RP-HPLC method for the simultaneous determination of five COVID-19 antiviral drugs: favipiravir, molnupiravir, nirmatrelvir, remdesivir, and ritonavir. There are three reported analytical methods for the quantitative determination of these studied anti-viral drugs The developed method offers several significant advantages, including simplicity, sensitivity, selectivity, and rapid analysis time. The chromatographic separation was achieved on a Hypersil BDS C18 column using a mobile phase of water and methanol (30:70 v/v, pH 3.0) with UV detection at 230 nm. All five compounds were well-separated with retention times under 5 min, making the method suitable for high-throughput analysis. The method was comprehensively validated according to ICH guidelines, demonstrating excellent linearity (r² > 0.999) in the concentration range of 10–50 µg/mL for all analytes. High trueness (99.59-100.08%) and precision (RSD < 1.1%) were achieved, along with adequate sensitivity as indicated by the low LOD (0.415–0.946 µg/mL) and LOQ (1.260–2.868 µg/mL) values. The method was successfully applied to the determination of these drugs in their pharmaceutical formulations with excellent recovery results, indicating the absence of interference from excipients. Environmental and practical assessments using a comprehensive five-tool evaluation approach revealed that the method demonstrates good environmental performance and excellent practical applicability. The green assessment tools (AGREE: 0.70, AGREEprep: 0.59, MoGAPI: 70%) collectively confirmed acceptable-to-good environmental impact, with particular strengths in solvent selection (methanol-water system), minimal sample preparation requirements, and isocratic elution mode. The blue assessment tools (BAGI: 82.5, CACI: 79) demonstrated excellent practical applicability, confirming the method’s suitability for routine laboratory implementation with standard instrumentation, reasonable costs, and efficient resource utilization. This multi-tool assessment approach, following current best practices in green analytical chemistry, provides robust evidence for the method’s environmental consciousness while maintaining the analytical rigor required for pharmaceutical quality control applications.

This work represents the first validated analytical method for simultaneous determination of these five COVID-19 antivirals, offering several key innovations: (1) Elimination of multiple separate analyses required by existing individual methods, reducing overall analysis time by approximately 75%; (2) Comprehensive green assessment using multiple evaluation tools, providing a holistic view of environmental impact; (3) Validation for pharmaceutical formulation analysis with statistical comparison to reported individual methods; (4) Short analysis time (< 5 min) compared to typical individual methods requiring 10–15 min each; and (5) Use of environmentally friendlier methanol-water mobile phase compared to acetonitrile-based systems commonly reported. Despite its advantages, the developed method has several limitations that should be acknowledged: (1) Limited to pharmaceutical matrices and may require adaptation for biological samples due to matrix complexity; (2) UV detection at 230 nm, while adequate for all analytes, may not provide optimal sensitivity for compounds with better absorption at other wavelengths; (3) Isocratic elution, while simpler, may not provide the flexibility of gradient methods for future method extensions; and (4) Method development did not employ design of experiments approaches, potentially missing optimal conditions.

Interestingly, several directions for future research emerge from this work. Several important directions for future research emerge from this work that would significantly expand the method’s applicability and impact. First, the method should be extended to environmental monitoring applications, particularly wastewater analysis, where these five antivirals are likely to co-occur due to their widespread use during the COVID-19 pandemic. This application would provide valuable insights for wastewater-based epidemiology, environmental fate assessment, and public health surveillance initiatives. Extension to wastewater matrices would require development of solid-phase extraction protocols for analyte preconcentration, validation of matrix effects in complex environmental samples, and potentially enhanced sensitivity to accommodate expected environmental concentrations. Second, the method could be extended to biological matrices such as plasma and urine to support pharmacokinetic studies and therapeutic drug monitoring of these antivirals. This would require additional validation for matrix effects and potentially modified sample preparation techniques. Third, the method could be adapted for environmental monitoring of these compounds in wastewater, which would contribute to understanding their environmental impact during pandemic situations. Fourth, the development of a stability-indicating version of this method, including forced degradation studies, would be valuable for shelf-life determination and quality control during long-term storage. Finally, future method development could benefit from implementing design of experiments approaches such as Box-Behnken design to further reduce solvent consumption and experimental trials during the optimization phase, as demonstrated in recent green analytical chemistry studies^36,37,50–55^. In conclusion, the developed method represents a significant contribution to the analytical toolkit for COVID-19 antivirals, offering a practical, environmentally friendly approach for their simultaneous determination in pharmaceutical quality control settings.

## Data Availability

The data presented in this study are available on request from the corresponding author.
